# Detection of the reaction intermediates catalyzed by a copper amine oxidase

**DOI:** 10.1107/S0909049510034989

**Published:** 2010-11-05

**Authors:** Misumi Kataoka, Hiroko Oya, Ayuko Tominaga, Masayuki Otsu, Toshihide Okajima, Katsuyuki Tanizawa, Hiroshi Yamaguchi

**Affiliations:** a School of Science and Technology, Kwansei Gakuin University, 2-1 Gakuen, Sanda, Hyogo 669-1337, Japan; b Physical Science Center for Biomolecular Systems Research, Kwansei Gakuin University, 2-1 Gakuen, Sanda, Hyogo 669-1337, Japan; c The Institute of Scientific and Industrial Research, Osaka University, 8-1 Mihogaoka, Ibaraki, Osaka 567-0047, Japan

**Keywords:** copper amine oxidase, AGAO, topaquinone, reaction intermediate, single-crystal microspectroscopy, X-ray crystal structure analysis

## Abstract

To reveal the chemical changes and geometry changes of active-site residues that cooperate with a reaction is important for understanding the functional mechanism of proteins. Consecutive temporal analyses of enzyme structures have been performed during reactions to clarify structure-based reaction mechanisms. Phenylethylamine oxidase from *Arthrobacter globiformis* (AGAO) contains a copper ion and topaquinone (TPQ_ox_). The catalytic reaction of AGAO catalyzes oxidative deaminations of phenylethylamine and consists of reductive and oxidative half-reactions. In the reduction step, TPQ_ox_ reacts with a phenylethylamine (PEA) substrate giving rise to a topasemiquinone (TPQ_sq_) formed Schiff-base and produces phenylacetaldehyde. To elucidate the mechanism of the reductive half-reaction, an attempt was made to trap the reaction intermediates in order to analyze their structures. The reaction proceeded within the crystals when AGAO crystals were soaked in a PEA solution and freeze-trapped in liquid nitrogen. The reaction stage of each crystal was confirmed by single-crystal microspectrometry, before X-ray diffraction measurements were made of four reaction intermediates. The structure at 15 min after the onset of the reaction was analyzed at atomic resolution, and it was shown that TPQ_ox_ and some residues in the substrate channel were alternated *via* catalytic reductive half-reactions.

## Abstract 2

Consecutive temporal analyses of enzyme structure have been performed during reactions in order to clarify the structure-based reaction mechanism. Four intermediate structures have been determined.

## 1. Introduction

To elucidate the mechanism of an enzyme reaction, it is important to characterize the enzymatic properties and then determine the enzyme’s function. Studies of reaction mechanisms have usually used biochemical and physicochemical methods such as spectroscopy. Using these techniques it is possible to determine the reaction mechanism by characterizing the amino acids that directly participate in the chemical reaction. However, the movements of side chains that cooperate with the reaction are important for promoting a smooth reaction. We have performed consecutive temporal analyses of enzyme structures during the reactions to clarify structure-based reaction mechanisms, including movements of the side chains that do not directly contribute to the chemical reactions.

Copper amine oxidases (EC 1.4.3.6) (CAOs) catalyze the oxidative deamination of primary amines to their corresponding aldehydes with the concomitant production of hydrogen peroxide and ammonia. In their active sites these enzymes contain a copper ion and a redox-active organic cofactor, 2,4,5-trihydroxyphenylalanine quinone (topaquinone, TPQ_ox_), which is a quinone cofactor that is covalently linked to the polypeptide chain. TPQ_ox_ is produced by the post-translational modification of the conserved precursor tyrosine residue in the presence of copper ion and molecular oxygen.

**Figure 1:**
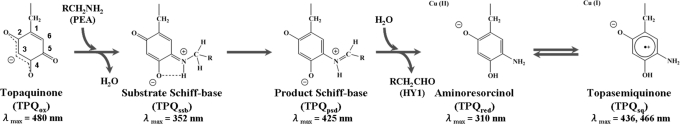
The proposed mechanism of the catalytic reaction of AGAO.

We have studied the CAO phenylethylamine oxidase from the Gram-positive bacterium *Arthrobacter globiformis* (AGAO). AGAO is a homodimer comprised of two subunits, each of which consists of 638 amino acid residues and has a molecular weight of about 70 kDa. The mechanism of TPQ_ox_ biogenesis, *i.e.* the conversion of precursor apo-AGAO to active holo-AGAO, has been identified by determining some of the major crystal structures of the biochemical reaction intermediates of AGAO (Kim *et al.*, 2002 ▶). A catalytic mechanism which has also been proposed by using biochemical and physicochemical methods is shown in Fig. 1 ▶ (Mure *et al.*, 2002 ▶). According to this proposal the reaction is resolved into two half-reactions. In the first reductive reaction the C5 carbonyl group of TPQ_ox_, its oxidized form, reacts with a phenylethylamine (PEA) and forms the substrate Schiff-base (TPQ_ssb_). The C1 proton from PEA is abstracted by a conserved catalytic base, an aspartic acid residue, and forms the product Schiff-base (TPQ_psb_). TPQ_psb_ is hydrolyzed, which gives rise to aminoresorcinol (TPQ_red_), its reduced form, and the release of the phenylacetaldehyde (HY1) product. TPQ_red_ equilibrates with topasemiquinone (TPQ_sq_) with simultaneously oxidizing the copper ion. In the subsequent oxidative half-reaction, TPQ_sq_ is reoxidized to TPQ_ox_ by molecular oxygen, and ammonia and hydrogen peroxide are produced. To identify the mechanism of the reductive half-reaction in this catalytic reaction, the four reaction intermediates were detected in the crystals and the structure at 15 min after the onset of the reaction (R15) was determined at atomic resolution.

## 2. Materials and methods

### 2.1. Enzyme purification and crystallization

The precursor apo-AGAO was overexpressed in *E. coli* cells and purified according to a previous procedure (Matsuzaki *et al.*, 1994 ▶). The purified solution of the precursor was dialyzed against 50 µ*M* copper sulfate buffer solution in order to convert it to the active holo-AGAO, and was then concentrated to 10 mg ml^−1^. Holo-AGAO was crystallized at 293 K by microdialysis against 1.05 *M* potassium sodium tartrate in 25 m*M* HEPES buffer, pH 6.8 (Kishishita *et al.*, 2003 ▶), in an anaerobic atmosphere in a glove box filled with N_2_ gas (∼99.9%). The dialysis buttons were transferred to a new reservoir solution containing 45% (*v*/*v*) glycerol as a cryoprotectant (Wilce *et al.*, 1997 ▶) for 1 day.

### 2.2. Trapping the reaction intermediates

Plate-form crystals with approximate dimensions of 0.4 × 0.3 × 0.1 mm were selected. The crystals were scooped using a thin nylon loop (diameter 0.3–0.4 mm) and then soaked in a 4 m*M* PEA solution for 1 to 120 min at 293 K in order to trap reaction intermediates. The crystals of the reaction intermediates were mounted on the loops and freeze-trapped by injection into liquid nitrogen.

### 2.3. Single-crystal microspectroscopy

The crystals of the reaction intermediates were analyzed at 100 K by single-crystal microspectroscopy to determine the stage of the catalytic reaction. The single-crystal microspectrophotometer system assembled by Dr Kawano, Riken Harima, comprised a deuterium tungsten lamp, Cassegrain mirrors, an optical fiber and a CCD-array spectrometer (Ocean Optics, PC2000). Absorption spectra of the crystals were recorded in the wavelength range 250–800 nm and corrected by an air-blank reference. These spectra were compared with spectra which were deconvoluted of chemical species in solution (Chiu *et al.*, 2006 ▶). From the results of previous studies the wavelengths that gave the maximum absorptions of the chemical species TPQ_ox_, TPQ_ssb_, TPQ_psb_, TPQ_red_ and TPQ_sq_ were 480, 352, 425, 310 and 436/466 nm, respectively.

### 2.4. Data collection and refinement

Diffraction data sets were collected at 100 K in a cold N_2_ gas stream with synchrotron X-radiation (λ = 1.000 Å) using an ADSC CCD detector in the stations BL38B1 and BL44B2 at SPring-8 (Hyogo, Japan). The data were processed and scaled using *HKL2000* (Otwinowski & Minor, 1997 ▶). The initial phases of the data sets were determined by molecular replacement by holo-AGAO (Protein Data Bank accession code 2cfd) as a search model using *MOLREP* (Vagin & Teplyakov, 1997 ▶). The initial models were refined using *REFMAC5* (Murshudov *et al.*, 1997 ▶; Vagin *et al.*, 2004 ▶). At the position of residue 382, the models for TPQ_ox_/PEA, TPQ_ssb_, TPQ_psb_ and TPQ_sq_/HY1 were built by using 2*F*_o_ − *F*_c_, *F*_o_ − *F*_c_ and omit maps. The refinement of the structure of R15 was completed and the structure has been deposited in the RCSB Protein Data Bank with the accession code 3AMO.

## 3. Results and discussion

### 3.1. Absorption spectra of the crystals

**Figure 2:**
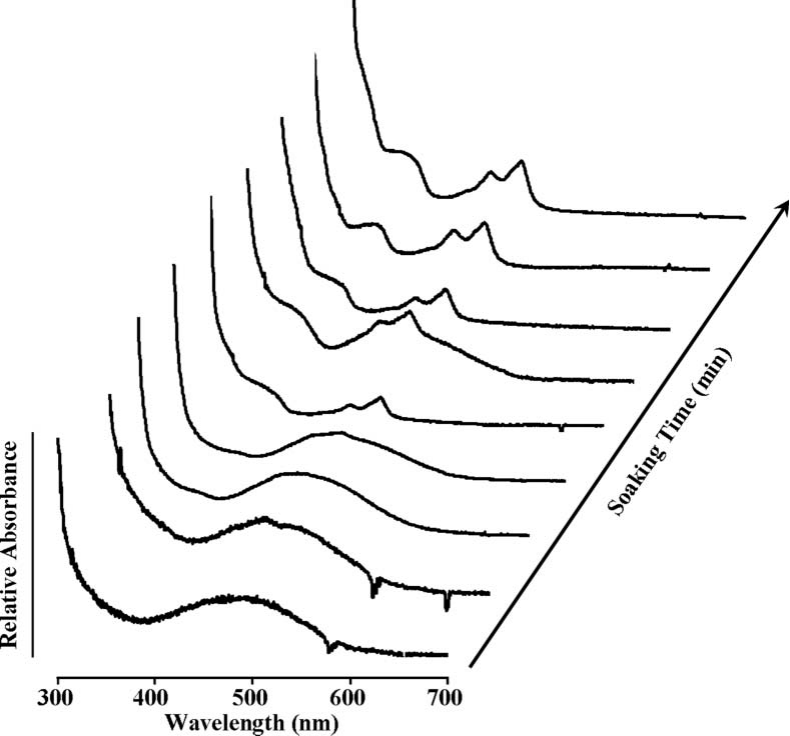
Results of the UV/vis absorption spectra changes during catalytic reaction of AGAO in each crystal with approximate dimensions of 0.4 × 0.3 × 0.1 mm. The crystals were soaked in PEA solution for 0, 1, 2, 5, 15, 30, 45, 60 and 120 min.

The UV/vis absorption spectra changes during the catalytic reactions of AGAO in each crystal are shown in Fig. 2 ▶. Based on the spectra of the crystals with deconvoluted absorptions, we found that the wavelength of the maximum absorption spectrum of the crystal soaked in PEA for 2 min was close to 352 nm, consistent with that of TPQ_ssb_. Two peaks, 436 and 466 nm, arose with the passage of time; peaks at 60 min, similar to 120 min, were 436 and 466 nm, which were consistent with those of TPQ_sq_. These results suggested that AGAO in crystals reacted with PEA during the time course from 2 to 60 min. In previous studies the reactive time in solution was 60 ms, whereas this time in crystals was 60 min. Therefore, the catalytic reaction time of AGAO in crystals was prolonged by as much as ∼6.0 × 10^4^ times the reaction rate in solution owing to the influence of the protein crystal field.

### 3.2. Structural change in the substrate channel

**Figure 3:**
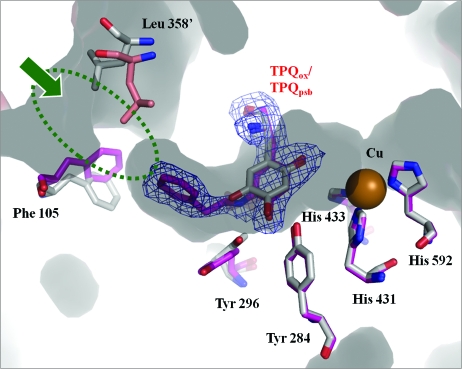
Superposed structures in the active site of crystals soaking in PEA solution for 0 (control) (gray) and 15 min (magenta). Based on the *F*_o_ − *F*_c_ omit map contoured at 2.4 s, it was revealed that the product Schiff-base (TPQ_psb_) was formed in this crystal. The substrate channel of AGAO is shown by the (green) circle. The entrance of the substrate channel (green arrow) was closed by Phe105 and Leu358′ of a neighboring subunit that moved as a cap.

The crystallographic statistics for R15 are shown in Table 1 ▶. We determined four crystal structures for the catalytic reaction intermediates of AGAO. Fig. 3 ▶ shows the superposition of chain B of R15 with the structure at 0 min (control). Based on the *F*_o_ − *F*_c_ omit map contoured at 2.4σ the electron density map of the TPQ_ox_ position was connected to that of the PEA position. We concluded that the product Schiff-base (TPQ_psb_) was formed as a result of fitting models for TPQ_ssb_ and TPQ_psb_ to the electron density map. This result almost corresponded to the ratio of the time change of the amount of TPQ_psb_ in the crystal to that in solution from previous work by using the mutant enzyme (Chiu *et al.*, 2006 ▶). We also found conformational changes of the amino acid residues in the active site, Phe105, and Leu358′ of a neighboring subunit that moved as a cap for the substrate channel.

In this study we could reveal the structural changes through an enzymatic reaction pathway not only for the active residues and a cofactor but also for the residues that did not directly participate in the chemical reaction. The detailed structures and the reaction mechanism based on the intermediate structures will be discussed elsewhere.

## 4. Conclusion

We have detected the reaction intermediates of AGAO in crystals and confirmed these by two methods: single-crystal microspectroscopy and X-ray crystal structure analysis. From the measurements of UV/vis absorption spectra of the crystals it was found that the two peaks for TPQ_sq_ arose during the time course of the experiment, but the spectra for the reaction intermediates could not be observed. However, using X-ray crystallography we succeeded in trapping and determining the reaction intermediates. Our results suggest that the reaction intermediates came to exist as a mixture, as the rates of reactions that were controlled in the crystals by substrate diffusion were not completely synchronized between the surface and the core, and because the reaction rate was dependent on each elementary reaction. In addition, the radiating beam positions for both single-crystal microspectroscopy and X-ray did not match entirely. Therefore, it is suggested that the absorption peak of a chemical species that had a small absorption was hidden by that of a chemical species that had a large absorption. If these experiments are carried out by irradiating the entire region using a smaller crystal or a microcrystal, the results from single-crystal microspectroscopy would correspond to X-ray crystal structure analysis. However, the detection of a significant electron density map for a reaction intermediate suggests that the reaction intermediate accumulates during that time. In conclusion, the results obtained here provide quantitatively sufficient information to trace the structural changes of a reaction.

The chemical reaction rate in a protein crystal generally becomes slow owing to limited protein motion under the influence of a protein crystal field. In this study the rate of the catalytic reaction of AGAO in crystals was 6.0 × 10^4^ times slower than that in solution. The reactions after soaking and trapping intermediates using cryogenic temperatures could become a general method for determining the intermediate structures of other proteins.

## Figures and Tables

**Table 1:** Statistics of data collection and crystallographic refinement of the crystal of R15 Figures in parentheses indicate the value for the highest-resolution shell (2.18–2.10 Å).

Unit-cell dimensions	
*a* (Å)	190.87
*b* (Å)	63.64
*c* (Å)	157.56
β (°)	116.82
Space group	*C*2
Total number of observations	631254
Total number unique reflections	93540 (9425)
Resolution (Å)	50.00–2.10 (2.18–2.10)
Completeness (%)	94.8 (96.1)
*R*_merge_ (%)†	7.0 (36.3)
Multiplicity	6.8 (5.9)
*I*/σ(*I*)	32.8 (6.5)

Refinements statistics	
*R*_work_ (%)‡	18.9
*R*_free_ (%)§	24.5
Average *B*-factors (Å)	28.1
R.m.s. deviation from ideal values	
Bond lengths (Å)	0.021
Bond angles (°)	1.9

† *R*_merge_ = Σ_*h*_Σ_*i*_|*I*_*h*,*i*_ − 〈*I*_*h*_〉|/Σ_*h*_Σ_*i*_ *I*_*h,i*_.

‡ *R*_work_ = Σ||*F*_o_| − |*F*_c_||/Σ|*F*_o_|.

§ *R*_free_ = *R*_work_ for approximately 5% of the reflections that were excluded from the refinement.
